# Supplementary material for: Molecular Signatures in Swine Innate and Adaptive Immune Responses to African Swine Fever Virus Antigens p30/p54/CD2v Expressed Using a Highly Efficient Semliki Forest Virus Replicon System
Source: Int J Mol Sci. 2023 May 26;24(11):9316. doi: 10.3390/ijms24119316 (PMC10253217; doi:10.3390/ijms24119316)
Supplement: Supplementary file 1 [file ijms-24-09316-s001.zip › supplementary Figures S1-S5.pdf]

Figure S1

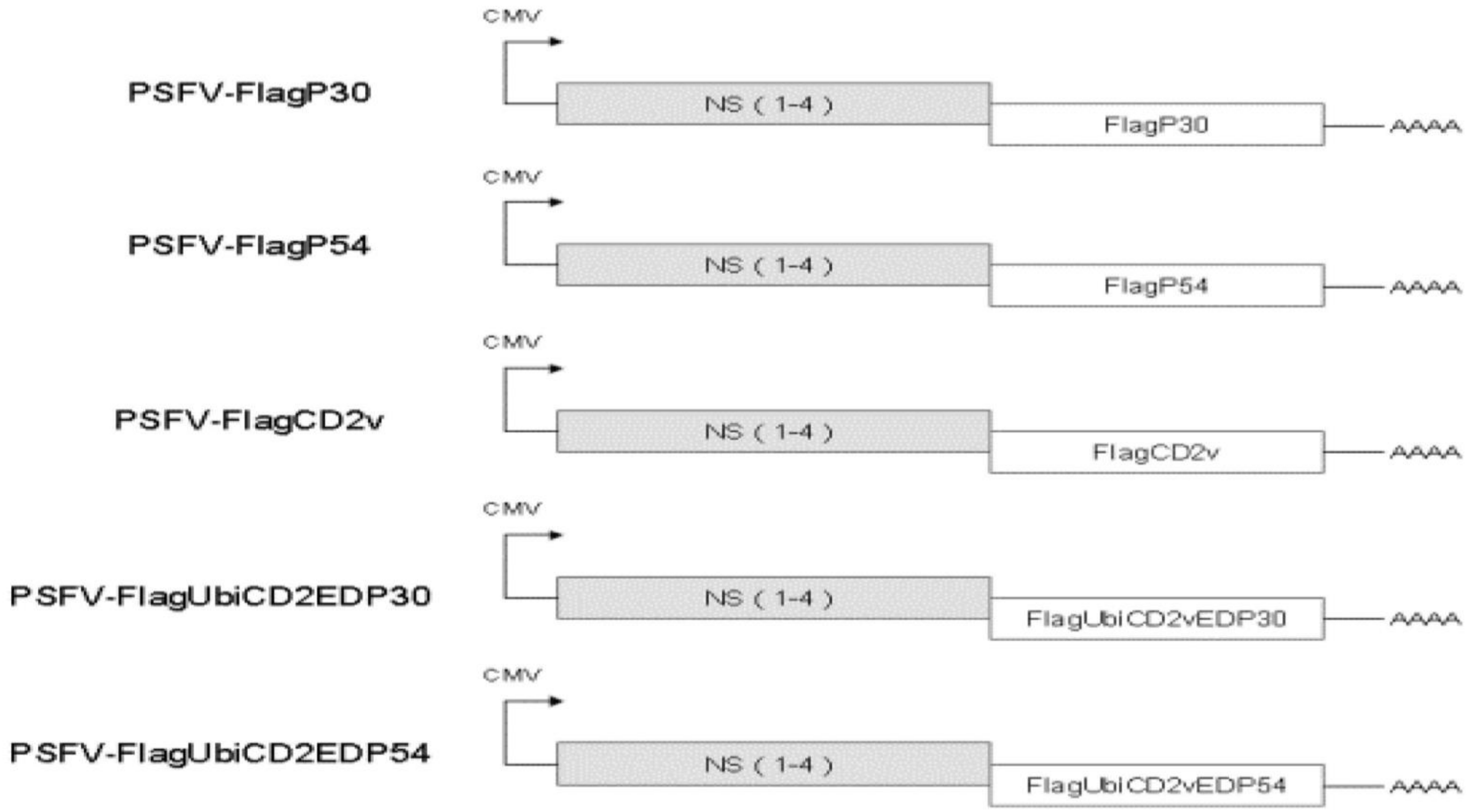

Figure S1. Plasmid construction

Diagrams illustrating the construction of plasmids SFV-Flagp30, SFV-Flagp54, SFV-FlagpCD2v, SFV-FlagUbiCD2vEDp30 and SFV-FlagUbiCD2vEDp54. The SFV transcriptase was constructed under the T7 promoter and antigen genes were inserted downstream of the S26 promoter of SFV.

Figure S2

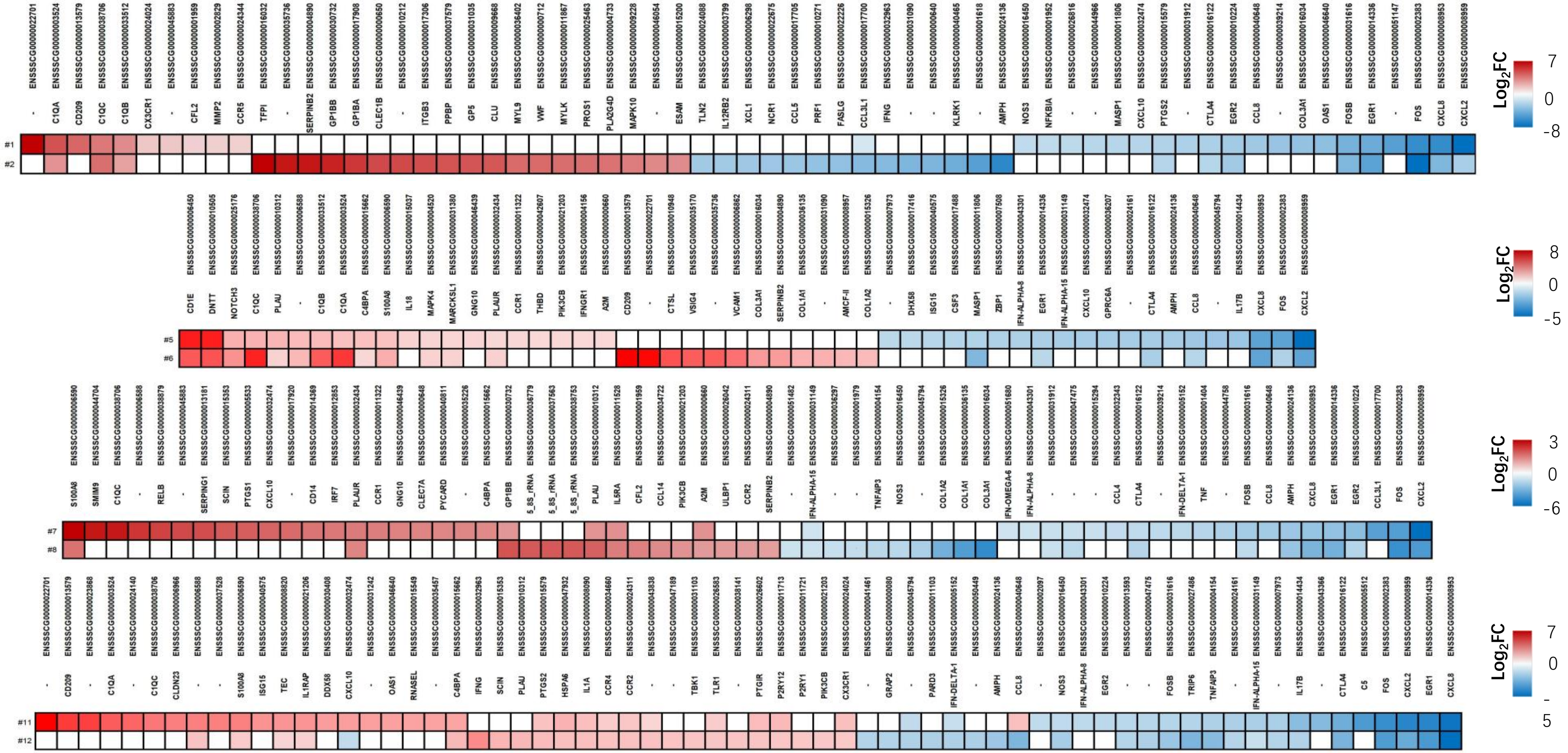

Figure S2. Top 20 DEGs up- and down-regulated in paired pigs.

Pair1 (#1 and #2) immunized with plasmid DNA without ubiquitin fusion; pair 2 (#5 and #6) immunized with IRP without ubiquitin fusion; pair 3 (#7 and #8) immunized with plasmid DNA with ubiquitin fusion; pair 4 (#11 and #12) immunized with IRP with ubiquitin fusion. Red: up-regulation; Blue: down-regulation.

Figure S3

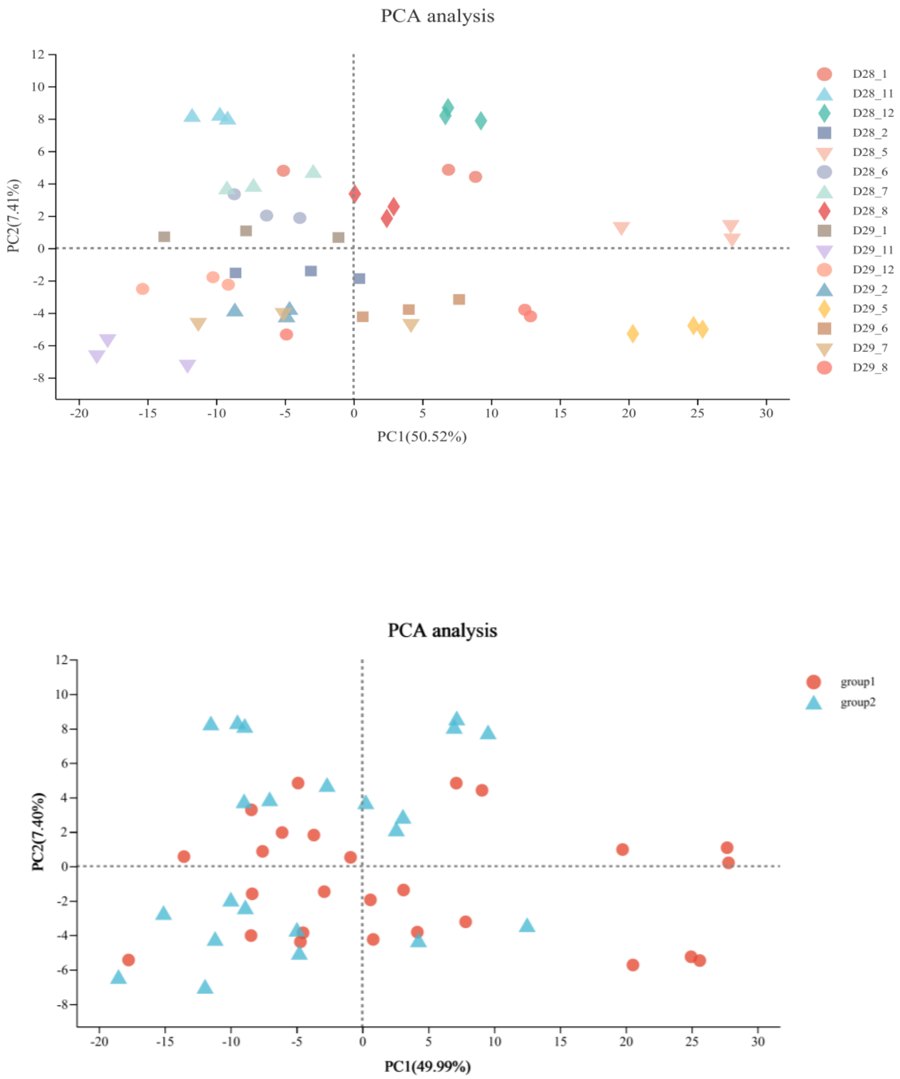

Figure S3. Principal component analysis (PCA) of transcriptomic data.

Top panel represents the plot from RNA sequence data collected on day 28 (D28) and day 29 (D29) from vaccinated individuals with three technological replicates, and the bottom panel represents the plot derived from data of group1 and group 2. The percentages on the axes indicate the values explained by each PCA. Samples obtained on D28 and D29 from different pigs are marked with different shapes and colors.

Figure S4

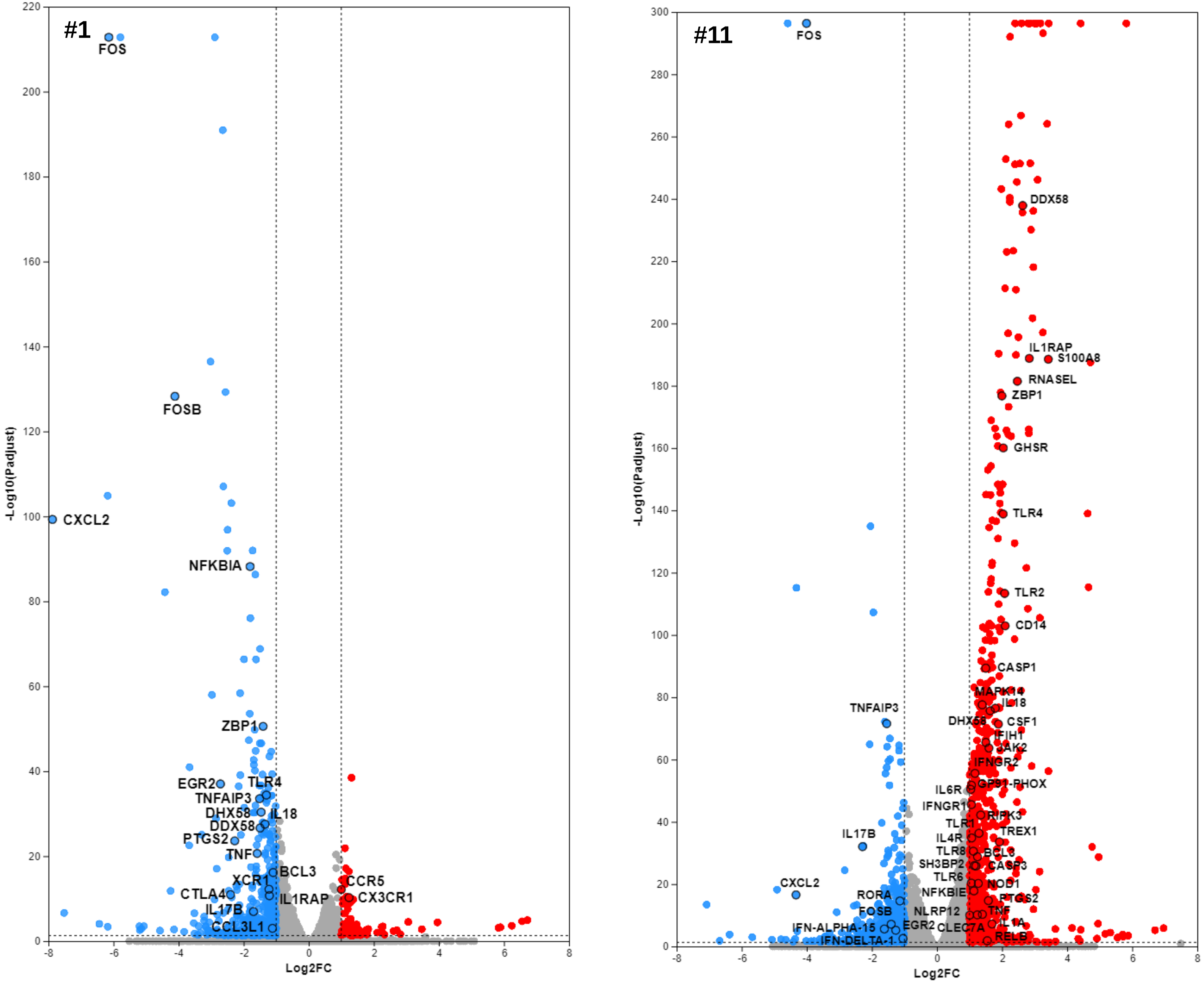

Figure S4. Volcano diagrams illustrating DEG expression patterns in #1 and #11 pigs.

The expression patterns of differentially expressed genes in the immune system in #1 and #11 pigs were elucidated. Blue, down-regulated genes; red, up-regulated genes; gray, genes without significant change. Y-axis, Padjust value; X-axis, log_2FC value. DEGs with Padjust <0.05 in functional groups annotated by KEGG were marked with gene names.

Figure S5

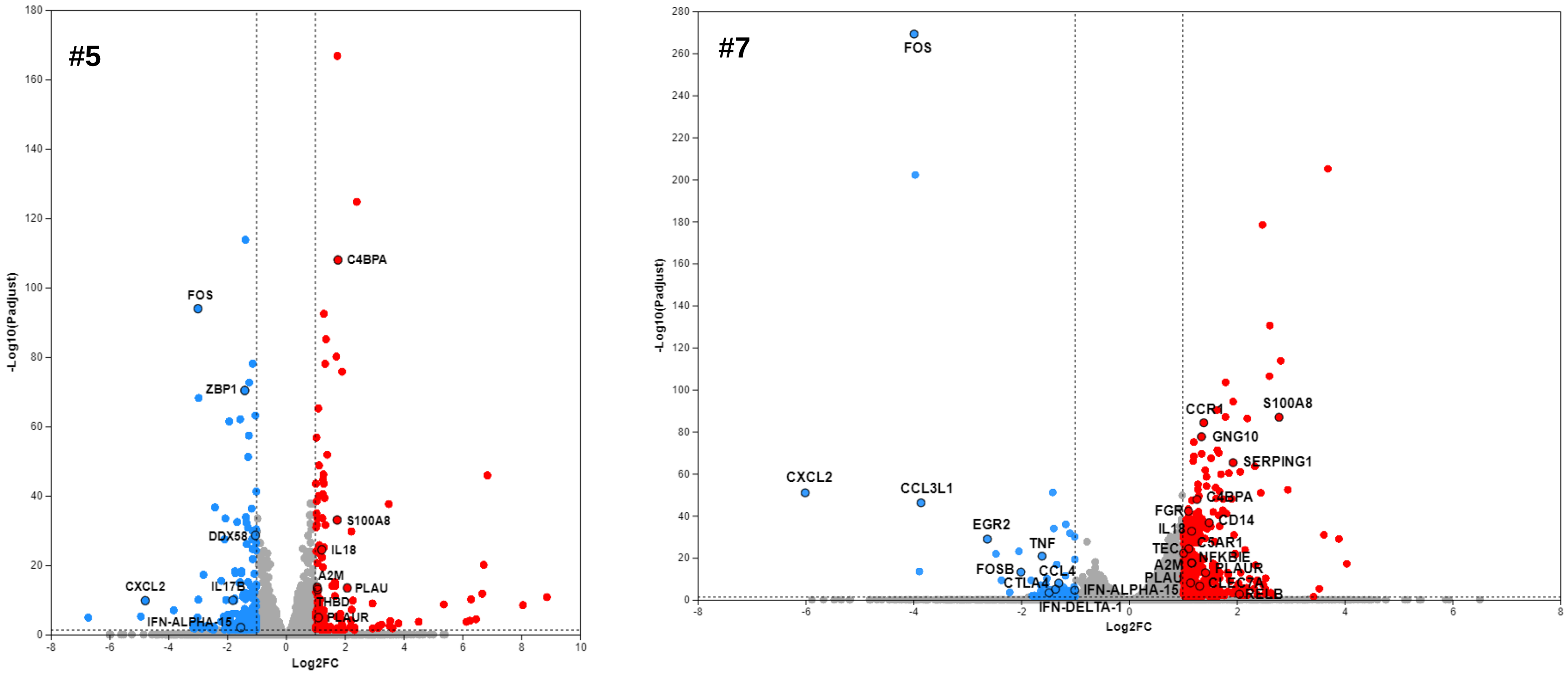

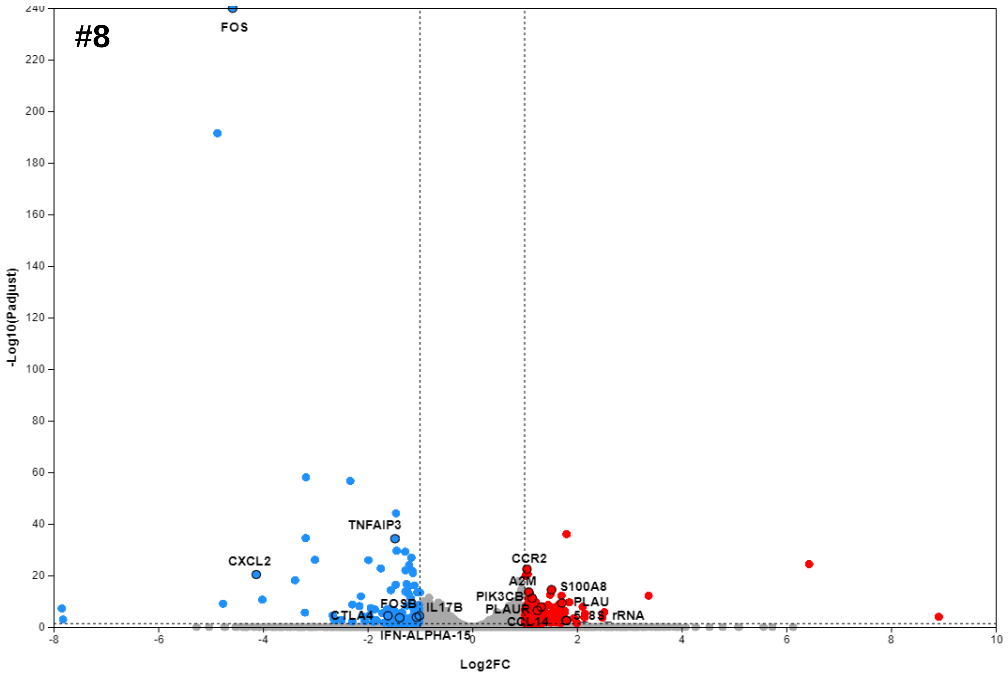

Figure S5. Volcano diagrams illustrating DEG expression patterns in #5, #7 and #8 pigs.

The expression patterns of differentially expressed genes in the immune system in #5, #7 and #8 pigs were elucidated as described in Figure S3.
